# Are epigenetic mechanisms and nutrition effective in male and female infertility?

**DOI:** 10.1017/jns.2023.62

**Published:** 2023-09-26

**Authors:** Kadriye Erdoğan, Nazlı Tunca Sanlier, Nevin Sanlier

**Affiliations:** 1 Department of Obstetrics and Gynecology, Health Sciences University, Etlik Zübeyde Hanım Women's Health Training and Research Hospital, Ankara, Turkey; 2 Department of Obstetrics and Gynecology, Ankara City Hospital, Ankara, Turkey; 3 Department of Nutrition and Dietetics, School of Health Sciences, Ankara Medipol University, Altındağ, Ankara 06050, Turkey

**Keywords:** Aging, Epigenetic, Female, Infertility, Male, Nutrition, 5mc, 5-methylcytosine, AMH, anti-Müllerian hormone, ART, assisted reproductive technique, CoQ10, coenzyme Q10, CpG dinucleotides, context of guanine, DMR, distinct methylated region, DNMT, DNA methyltransferase, FSH, follicle stimulating hormone, H2A, H2B, H3 and H4, nucleosomal core histones, HOXA10, HomeoboxA10, HPR, histone-protamine ratio, ICMART, International Committee for Monitoring Assisted Reproductive Technologies, ICSI, intracytoplasmic sperm injection, ICR, imprinted control region, IL-6, interleukin-6, IVF, in vitro fertilisation, lncRNA, long non-coding RNA, MAR, matrix attachment region, miRNA, micro-RNA, mRNA, coding RNA, MTHFR, methylenetetrahydrofolate reductase, ncRNA, non-coding RNA, NIFT, non-IVF fertility treatment, NTD, neural tube defect, OAT, oligo-astheno-teratozoospermia, P1, P2, potamine 1, potamine 2, PCOS, polycystic ovary syndrome, piRNA, piwi-interacting RNA, ROS, reactive oxygen species, SAM, *S*-adenosylmethionine, SHBG, sex hormone-binding globulin, siRNA, small interfering RNA, SNP, single nucleotide polymorphism, SNRPN, small nuclear ribonucleoprotein polypeptide N, TP1, TP2, transition proteins, UMI, unexplained male infertility, VDR, vitamin D receptor

## Abstract

This review discusses epigenetic mechanisms and the relationship of infertility in men and women in relation to parameters pertaining to nutrition. The prevalence of infertility worldwide is 8–12 %, and one out of every eight couples receives medical treatment. Epigenetic mechanisms, aging, environmental factors, dietary energy and nutrients and non-nutrient compounds; more or less energy intake, and methionine come into play in the occurrence of infertility. It also interacts with vitamins B12, D and B6, biotin, choline, selenium, zinc, folic acid, resveratrol, quercetin and similar factors. To understand the molecular mechanisms regulating the expression of genes that affect infertility, the environment, the role of genotype, age, health, nutrition and changes in the individual's epigenotype must first be considered. This will pave the way for the identification of the unknown causes of infertility. Insufficient or excessive intake of energy and certain macro and micronutrients may contribute to the occurrence of infertility as well. In addition, it is reported that 5–10 % of body weight loss, moderate physical activity and nutritional interventions for improvement in insulin sensitivity contribute to the development of fertility. Processes that pertain to epigenetics carry alterations which are inherited yet not encoded via the DNA sequence. Nutrition is believed to have an impact over the epigenetic mechanisms which are effective in the pathogenesis of several diseases like infertility. Epigenetic mechanisms of individuals with infertility are different from healthy individuals. Infertility is associated with epigenetic mechanisms, nutrients, bioactive components and numerous other factors.

## Graphical abstract

**Figure:**
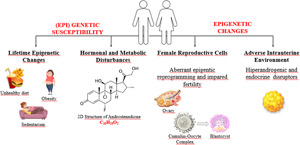

## Introduction

Having a child is arguably a situation that bears a multifaceted issue with its psychological, economic and societal dimensions in a fair number of cultures, and it is deemed crucial assuming that it provides individuals with privilege and prestige in their society. Parents tend to teach their children about gender roles and the accepted social norms in their society from the moment they are born. Individuals who grew up with these cultural norms perceive infertility as the inability to fulfil the requirements of their masculinity for men, and the inability to meet the expectation of motherhood in society for women^(1)^. From a scientific overview, infertility, one of the most common reproductive health disorders, is portrayed by the World Health Organization (WHO) as well as the International Committee for Monitoring Assisted Reproductive Technologies (ICMART) as the lack of clinical pregnancy proceeding 12 months or more of regular and unprotected sexual intercourse. Worldwide, the prevalence that is pertinent to infertility in partners who are at reproductive ages is between 8 and 12 %^(2)^. The rates are 6⋅9–9⋅3 % in the countries classified as developing, and 3⋅5–16⋅7 % in the ones counted as developed^(3)^. It has been reported that approximately one in eight couples seeks medical treatment after failing to conceive for a year^(4)^. When it comes to addressing the matter through the available methods, it would be fair to say that assisted reproductive techniques (ARTs) are most commonly used in the medical treatment of infertility. The technique in which infertile couples have the highest rate of having a baby with ART is *in vitro* fertilisation (IVF)^(5)^. Howbeit, surgery, drug therapy and treatment with IVF or other ART for infertility do not yield pregnancy and/or live birth all the time^(6)^. Despite the perception that the cause of infertility mainly belongs to the female partner, it is indeed a fact that it is evenly distributed between the sexes. According to various studies, approximately 20–35 % of infertility cases are female, 20–30 % are male, 25–40 % are due to combined problems in both parts and 10–20 % have unexplained infertility without an identifiable cause. The type of unexplained infertility is known as idiopathic^(7–9)^. While 80 % of cases that pertain to infertility may be linked to circumstances like endometriosis or polycystic ovary syndrome (PCOS), 20 % remain non-explained. The high incidence of infertility may depend on many parameters, e.g. age, environment, lifestyle alongside health status. In light of all the aforementioned reasons, the present study will attempt to concentrate on the epigenetic dimensions of infertility in both men and women and the risk factors linked to nutrition.

As it has been mentioned in earlier parts of the paper, female fertility is the determinant of the gender role in society in addition to being a biological fact, which constitutes a constraint for specific groups such as relatively elder women. Being older than 35 years of age indeed increases infertility risk^(10)^. As for males, men's testosterone levels decline with age, and sperm motility and semen volume also decrease after the age of 35. Moreover, DNA damage in sperm increases significantly after the age of 40, and both the motility and viability of sperm are reduced^(11)^. The changes are called epigenetic marks; DNA methylation, remodelling of chromatin, histone tail modifications apart from mechanisms related to non-coding RNAs^(12)^. This takes over an eminent function in the monitoring of cellular processes such as gene expression levels, DNA–protein interactions, differentiation at cellular level, embryogenesis, aside from genomic imprinting^(13,14)^. Genomic imprinting, a phenomenon of epigenetic gene marking emerging in the germline, results in parental-specific occurrence of a tiny gene subset of mammals. Imprinting indeed owns a significant effect over normal mammalian development, fetal growth, metabolism as well as adult behaviour. Epigenetic marks of parental origin are structured when male and female gametogenesis take place, transferred over to the zygote via fertilisation, sustained during the course of development as well as adulthood, are deleted in primitive germ cells prior to the formation of novel marks^(15)^. As is known the epigenome alone does not encode genetic information, but is responsible for the expression of genes, does not control intergenerational inheritance, but can replicate itself from cell to cell. Epigenetic changes in gametes are likened to changes that occur in the four seasons of life, and gamete development can be evaluated in four stages: embryonic development, the process up to puberty and sexual maturity, adulthood and the aging process. This journey of gametes is full of epigenetic changes^(16)^. Epigenetic modification can be transmitted relatively stably during the process of cell proliferation. In recent years, it is thought that changing gene expressions through epigenetic mechanisms may cause several health problems^(17)^.

## Methods

A review of the line of the literature was conducted prior to June 2021 through the determined websites, i.e. MEDLINE, Embase, Web of Science, www.ClinicalTrials.gov, Cochrane Central, PubMed, Google Scholar, Science Direct and the WHO. The targeted papers were clustered referring to the existing information using some keywords: ‘infertility, epigenetics, genome, DNA sequence modification, gene expression, diet and infertility, dietary habits, semen parameters, dietary patterns, foods, nutrients, fertility and infertility’. The keyword combinations are resorted to again through the names of infertility, epigenetics and nutrient in family names. Sub-references of the articles identified relying on keywords were sought and these were scrutinised too. In reserach on infertility, epigenetics and nutrients, animal studies together with clinical human studies were explored. The said reviews as well as research meta-analyses constitute the framework of the current research.

### Epigenetic mechanisms and infertility

There are more than 200 cell types of different sort in the human body and each of these types possess the same copy of the genome. Howbeit, distinctive types of cells form different gene series that depend upon epigenetic regulation. Epigenetics examines mitotic and/or meiotic hereditary changes which influence gene expression with the lack of DNA sequence modification. The epigenome is thought to function as the second dimension of the DNA sequence that is highly effective in maintaining patterns that belong to cell type-specific gene expression^(12)^.

Since a cell's epigenome has great plasticity and can be reprogrammed, epigenetic modifications dynamically and reversibly control gene expression. Epigenetic reprogramming alters the fate of cells throughout development and adulthood. It is noteworthy that environmental factors play an important role in the creation and maintenance of epigenetic marks^(18)^.

### Male infertility

Epigenetic processes are influential in male fertilisation potential as well as sperm function. The appropriate functioning pertaining to epigenetic mechanisms, namely, ncRNAs DNA methylation, chromatin remodelling and histone tail modifications during gonadal as well as spermatogenesis development is essential for normal sperm production and function^(12)^. Speaking of settings of clinical sort, semen analysis is commonly used to diagnose the trend of male-related infertility, to wit, DNA fragmentation analysis as well as microscopic examination, which are not sufficient to explain all events. However, spermiograms belonging to infertile patients’ normozoospermic cannot be distinguished from those of fertile individuals, which is often insufficient for the diagnosis of unexplained male infertility (UMI). Nevertheless, recent advances in sequencing technologies are promising as they can help us identify why some couples experience idiopathic infertility^(19)^. Remodelling of chromatin, residual histone alterations alongside DNA methylation reflect the key epigenetic shifts that occur at the sperm RNAs, and level of sperm seems to play an important role too^(20)^. Methylenetetrahydrofolate reductase (MTHFR) enzyme activity and its product *S*-adenosylmethionine (SAM) play a role in sperm evolution, morphology and motility. It has been observed that MTHFR enzyme is an enzyme involved in folic acid metabolism and its activity may be an important factor in spermatogenesis^(21)^. In a study, it was determined that MTHFR hypermethylation gene promoter occurs often in sperm obtained from infertile individuals, and it is relatively more common in males with a history of spontaneous abortion than in men without a history of spontaneous abortion, and it oftentimes impact the entire population of sperm. This result indicates a new male parameter linked with spontaneous abortion infertility. For this reason, MTHFR hypermethylation gene promoter appears to emerge as a new putative risk factor in the spontaneous abortion aetiology^(22)^. In another study, abnormal methylation was found within the gene promoters related to imprinting, spermatogenesis as well as defense system of antioxidant sort in the sperm of patients having impaired sperm DNA integrity status^(23)^.

### Female infertility

According to the results of a study, it is known that epigenetic deviation, which is an eminent factor of biological sort, causes diseases and gene dysregulation. It has been shown that abnormal expression of the *HomeoboxA10 (HOXA10)* gene and constructions of epigenetic sort are effective in the pathophysiology of endometriosis^(24)^. A fair number of studies like the work of Taylor and Gui have also reported that the body growth regulator *HOXA10* gene shows aberrations within the endometrium of females having endometriosis. Located on chromosome 7p15.2, this master regulatory gene is the member of a larger family pertaining to DNA-binding transcription parameteres sharing an utterly conserved 183-nucleotide sequence encoding a 61 amino acid homeodomain. *The HOXA10* homeobox is known to control uterine organogenesis throughout the development of the embryo as well as endometrial differentiation of functional sort in adults^(25,26)^. Expression of the *HOXA10* gene belonging to fertile women who are healthy individuals is cycle dependent. Levels of *HOXA10* messenger RNA (mRNA) boost significantly during the stage pertaining to mid-secretory, a condition that coincides with implantation of embryo, peak differentiation at histological level as well as elevated systemic estrogen along with levels of progesterone^(24)^. Expression at higher levels pertaining to *HOXA10* in the endometrium is indispensable for the decidual transformation of endometrial stromal cells owing to implantation of embryo. A defect occurring in the expression of *HOXA10* and its regulation which results in miscarriages of recurrent sort as well as infertility yield impaired implantation and decidualisation^(27)^.

Most infertility pertaining to women is caused by PCOS, endometriosis as well as infertility of non-explained being^(28)^. PCOS is among the main reasons behind women infertility worldwide. We know that susceptibility to PCOS can be inherited not only by genetic alleles but also by epigenetic changes and developmental programming^(29)^. Environmental factors caused by hormonal and metabolic disturbances bring about (epi)genetic susceptibility to the development of PCOS throughout life. PCOS development and clinical manifestations are directly influenced by hormonal and environmental changes, so epigenetic alterations might also be influential in PCOS outcomes^(18)^.

### DNA methylation

Methylation of DNA is an epigenetic marker that is frequently inquired into. This alteration of epigenetic sort is required for the development of women and men gametogenesis. Within the mammalian genome, DNA methylation emerges mainly in the frame of guanine (CpG dinucleotides) and cytosine at the fifth position pertaining to the cytosine bases. This is called 5-methylcytosine (5mc)^(19)^. These dinucleotides are defined as methylated regions in a different fashion (a.k.a. DMRs (distinct methylated regions)), which are usually located closer to regions that are known as gene regulatory like the promoter. DNA methylation covers the methylation pertaining to non-imprinted as well as to those that are imprinted, methylation that is pertinent to elements of repetitive sort, and DNA methylation at global levels^(20)^.

Methylation of cytosine at DMRs inhibits the process that pertains to the binding of transcription factors to the locations that are considered regulatory towards the relevant genes, verging on silencing or transcriptional inactivation. On the contrary, hypomethylation of regulatory regions is attributed to enhanced gene expression^(12)^. DNA methyltransferases (DNMTs) are named DNMT3A, DNMT1, DNMT3L and DNMT3B^(30)^. One of the enzymes that maintain DNA methylation throughout DNA replication is DNMT1. The lack of DNMT1 results in aberrations throughout spermatogenesis as well as in the loss of methylation chiefly in imprinted genes of paternal sort. The enzymes DNMT3B, DNMT3L, along with DNMT3A take part in the DNA methylation throughout germ cell development at the embryonic phase. All of the DNMTs are required for appropriate spermatogenesis to occur^(31)^.

Sperm DNA methylation is linked to sperm changes as well as to infertility. Genes frequently linked with male infertility contain defects pertaining to DNA methylation of the imprinting genes mesoderm-specific transcript (*MEST)* alongside *H19* as well as those of non-imprinting sort MTHFR. Methylation regulates expression in relation to imprinted genes through a pivotal process, which causes an occurrence of the allele pertaining to maternal or paternal side. Once maternal/paternal alleles are demethylated with fertilisation, genes that are imprinted retain methyl markings that pertain to the parental genome^(32)^.

Imprinted genes indicate parent-specific activity and are functionally haploid. Therefore, they become vulnerable to epigenetic dysregulation. To put it more specifically, both maternal and paternal alleles are subject to demethylation following the fertilisation stage. After that, reprogramming of genetic sort takes place within the embryo, consisting of new specific methylations. Genes that are imprinted get isolated from reprogramming of epigenetic sort following the fertilisation making transmission of abnormal methylation patterns to the relevant offspring possible^(20)^. *Rasgrfl, Igf2/H19, Zdbf2* and *Dlk-Gtl2* are genes that are paternally methylated within spermatozoa. The *Model H19* gene is baled to encode a 29k protein as well as a cytoplasmic RNA of untranslated sort participating in the transport/synthesis and RNA processing pertaining to a protein. *H19 DMR* is not methylated on the allele that is maternal. For this reason, the expression of *H19* is made possible and insulin-like growth factor II (*IGF2*) gene becomes suppressed. Yet, methylation pertaining to the *H19 DMR* allele of paternal sort blocks *IGF2* gene expression^(12)^. In normozoospermic males, *H19* gene methylation gets linked to reactive oxygen species (ROS) levels as well as factors that pertain to semen. According to a meta-analysis study analysing aberrations of sperm DNA methylation pertaining to imprinted genes, *MEST*, and levels of small nuclear ribonucleoprotein polypeptide N (*SNRPN) NAME DMR* methylation are considerably higher in idiopathic infertile males when compared to fertile males. *H19 DMR* methylation levels were found to be lower in infertile men than in fertile men^(12,33)^. According to this, hypermethylation of the *SNRPN* and hypomethylation pertaining to the *H19 imprinted control region (ICR)* are associated with infertility. The risk of this relationship increases with the habit of smoking. Furthermore, drinking alcohol is ascribed to DNA methylation within regions of regulatory sort pertaining to the imprinted gene *H19* in human and mouse spermatozoa. However, the influence over alcohol-induced DNA methylation on fertility should be investigated further^(19,33)^. A global DNA hypomethylation occurs with ARTs. Moreover, risk of boosted sort that pertains to imprinted disorders has been recorded following the procedures in question. This has led to the emergence of the argument that ART might lead to a loss in methylation. In a study, a global DNA methylation that is considerably distinctive was identified within sperm obtained from individuals having oligo-astheno-teratozoospermia (OAT) when compared to the controls and showed the said epigenetic abnormalities can reduce human fertility^(20)^.

### Chromatin reorganisation

By the rearrangement of sperm chromatin, spermatozoa pack a large amount of DNA within a minute nucleus. Protamines are known as small proteins that are unique to spermatozoa. Thanks to the existence of the passage pertaining to protamines through histones, sperm DNA occupies smaller space within the nucleus. Thusly, it causes a condensation that is pertinent to the sperm nuclei which is of tighter sort supporting the increase in sperm motility. What is more, protamination prevents the sperm genome from degradation as well as from oxidation and molecules of harmful sort found within the women reproductive system^(12)^. In the final phases pertaining to male gametogenesis (spermiogenesis), motile spermatozoa emerge from haploid round spermatids. Chromatin gets subject to a drastic remodelling throughout which almost all of (90–95 %) of the *H2A*, *H2B*, *H3* and *H4* (nucleosomal core histones) are replaced by transitional proteins first and by protamines afterwards^(19)^.

Spermatozoa are known to be cells of excessively specialised sort. Through the course of spermatogenesis, almost all of the chromatin histones (90–95 %) are exchanged with protamines, which are nuclear proteins that are tiny and arginine-rich. The said process occurring, which is expected for spermatozoa, provides considerable DNA compaction, reduced vulnerability towards outside factors and constitutes a gene silencing mechanism. In the earlier phases of protamination, acetylation of histones increases first, which supports enzyme DNA topoisomerase action, suceeded by the replacement of *histones having transition proteins (TP1 and TP2)*. The said DNA-binding proteins trigger the removal as well as the subsequent replacement pertaining to histones by equally expressed *protamine 1 (P1)* and *protamine 2 (P2)*^(12)^.

DNA organisation at three levels may be observed after sperm chromatin protamination: (1) toroidal structures shaped by protamines (90–95 %), (2) nucleosomes (5 %−10 %) taking part in the chief stages of the development of embryo alongside (3) the so-called matrix binding sites. DNA segments do not contain toroidal structures or nucleosomes. *MARs (Matrix Attachment Regions)* warrant structural support to chromatin act as promoters in the paternal pronuclear formation after fertilisation and contribute to normal embryogenesis. Consequently, scientific evidence has shown abnormalities pertaining to protamine content can impact epigenetic information transmitted by DNA of paternal sort. Accordingly, the state of sperm protamination also affects the results of ART^(20)^.

### Histone alterations

Modifications of histone negatively or in a positive fashion affect the binding of regulatory factors to DNA, resulting in diminished or escalated activity pertinent to gene and expression. Specific modifications, e.g. acetylation pertaining to *H3* and *H4*, methylation that pertains to H3K4, as well as the ubiquitination belonging to H2B increase gene expression within testicular tissue. On the contrary, methylation pertaining to *H3K27* along with *H3K9* and ubiquitination pertaining to *H2A* result in the silencing of gene expression. It has been suggested that *H3K4* and H3K27 methylation stimulate the inactivation as well as the activation of gene expression^(20,34,35)^. Histones that are retained are visible in gene clusters of imprinted sort and therefore protamination as well as changes within residual histones may be the common cause of paternal infertility. Correspondingly, research conducted on 291 ART cycles identified the function pertaining to *histone-protamine ratio (HPR)* on embryonic development and ART outcomes. The blastocyst formation rate for *HPR*, ranging from 6 to 26 %, was considerably higher (87⋅8 %) than what was achieved through *HPR* >6 % (74⋅6 %) or <6 % (71⋅2 %). Thereupon, *HPR* appears to have an effect on embryo development. Based on this evidence, protamine-free sperm apart from residual histone abnormalities need to be used in programmes of ART^(20)^.

### Sperm-derived coding and non-coding RNA molecules

Studies have shifted more towards elucidating the functions of these RNAs. Albeit transcriptionally quiescent, sperm are indicated to involve non-coding (ncRNA) and coding (mRNA). RNA molecules that have been declared as essential for epigenetic inheritance in a wide spectrum, earlier development as well as spermatogenesis. Sperm carry thousands of different RNAs, encompassing non-coding (ncRNA) (antisense RNA, tiny interfering RNA (siRNA), micro-RNA (miRNA), long non-coding RNA (lncRNA), together with piwi-interacting RNA (piRNA)) alongside coding (mRNA) RNAs, and through different mechanisms, it takes part in the modulation of gene expression via interrupting mRNA translation^(20)^. Analysis of transcriptomic sort pertaining to sperm from males with idiopathic infertility (normozoospermic), asthenozoospermia (reduced motility) and known fertility spotted diverse profiles of RNA among patient cohorts and emphasised the potential role belonging to the molecules in paternal fertility^(19)^. Environmental toxicants have been shown to affect testicular disease and the epigenetic transgenerational inheritance of male infertility. It involves epigenetic changes in the germline (e.g. sperm) to affect the epigenome and transcriptome of early embryonic stem cells. As the male population has a decrease in sperm count and a dramatic increase in infertility, observations suggest that testicular disease may be an important component of male infertility aetiology of promoting epigenetic transgenerational inheritance^(36)^. In another study, methyloma analysis of individual blastocysts compared with fertile controls unearthed considerable changes at 6609 CpG sites linked to long-term infertility (≥60 months). In sum, long-term infertility is associated with a modified methylome in euploid blastocysts with specific concentration on regulation pertaining to genomic imprinting, which is compared to aided reproductive technologies alone^(37)^. *H19* also encodes for non-coding RNA^(38)^.

### Treatments related to genetics and epigenetics of infertility

The use of ARTs covers IVF. Other treatments of non-IVF fertility (NIFTs) sort, encompassing controlled ovarian stimulation and ovulation induction with intrauterine insemination, provide 4⋅6 % of live births^(28,39)^. Pregnancy outcomes of adverse nature are ascribed to the usage of ART. A meta-analysis showed that the risks of pregnancy-induced hypertension, placenta previa, placental abruption, gestational diabetes mellitus, preterm birth, postpartum haemorrhage, small for gestational age, low birth weight as well as perinatal mortality are increased. Infertility of maternal sort derives from several aetiologies, which may impact subsequent placentation as well as implantation, resulting in outcomes of adverse sort such as PCOS, unexplained infertility, endometriosis, along with placental dysfunction. PCOS is responsible for 0⋅70 % of all ovulatory dysfunction cases accounting for 27 % of infertility reportings. PCOS is a disorder of endocrine metabolic sort that affects 5–15 % of females^(28)^. Even after the attainment of ovulation, females having PCOS appear to have reduced rates of cumulative pregnancy compared to subfertile populations^(28,40)^ of another sort. Endometriosis constitutes a condition depicted typically by stroma outside of the uterine cavity and endometrial glands, leading to infertility, dysmenorrhoea as well as pelvic pain. It impacts approximately 10 % of women who are at a reproductive age. As a result, it has been recommended that 25−50 % of infertile females may be with endometriosis and 30−50 % of females with endometriosis may be infertile. Epigenetic and genetic effects pertaining to infertility aetiologies alongside effects of environmental sort belonging to fertility treatments upon epigenetics affect placentation and implantation. This results in shorter as well as longer-term maternal and fetal/child outcomes that are of adverse sort^(28)^.

Single gene disorders resulting in primary sterility as well as disease are detected. That said, a smaller ratio pertaining to infertility aetiologies are contributed by these specific disorders and failed to explain many of the multifactorial causes^(41)^. More recently, single nucleotide polymorphisms (SNPs) have been ascribed to diseases that cause infertility^(28,42)^.

**Fig. 1.**
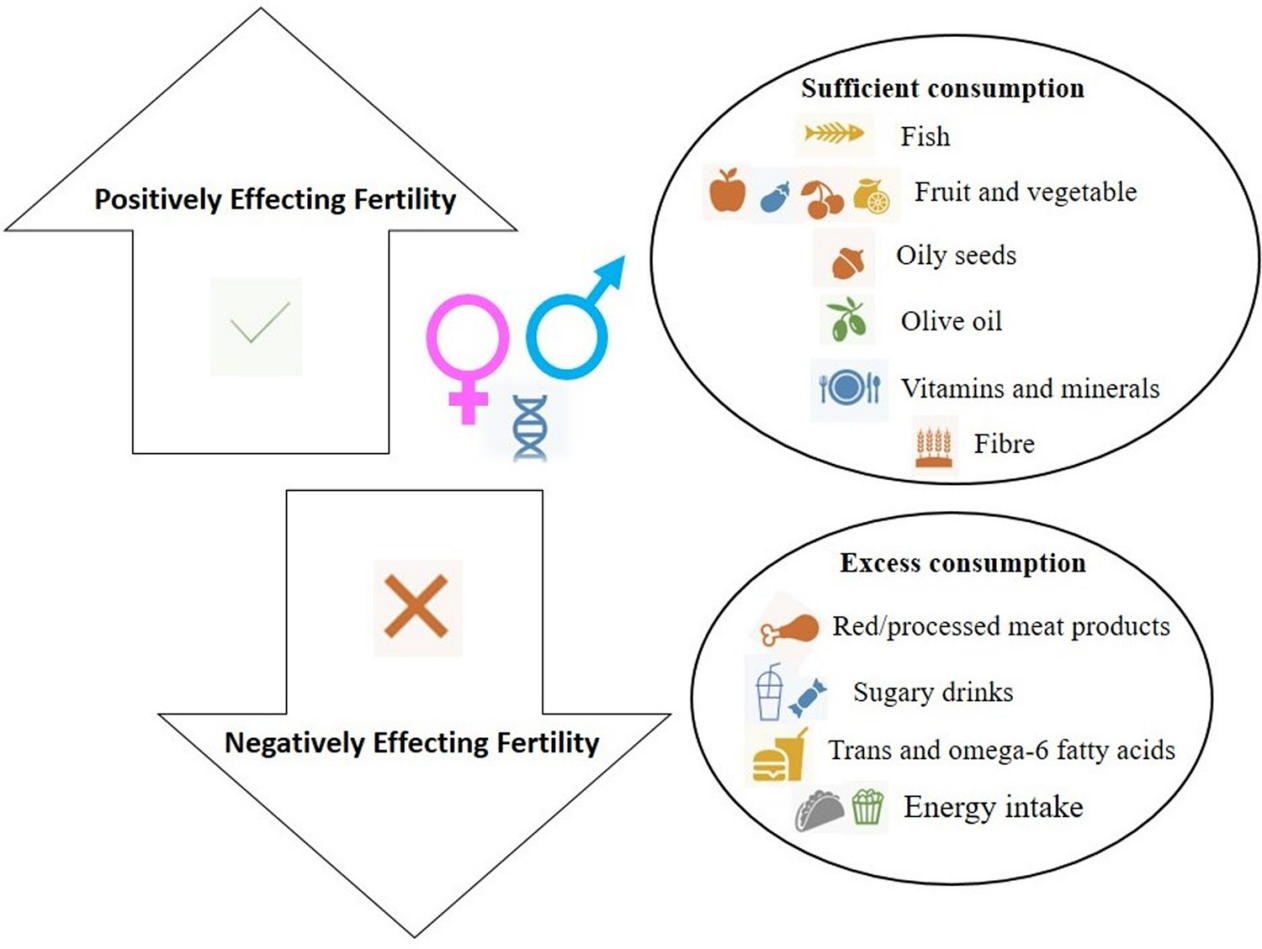
Characteristics and suggested modification of diet that adversely affects fertility.

Epigenetic modifications are inherited that cannot be caused by the alterations in the DNA sequence. Diseases are impacted by environmental factors and genetic variability^(38,43)^. These non-coding mechanisms of gene regulation contain non-coding regulatory elements and DNA methylation. They regulate gene expression patterns by altering DNA accessibility and chromatin structure^(28)^. Short non-coding RNAs, containing lncRNAs as well as miRNAs, influence the overall gene expression in the transcriptome. Longer non-coding RNAs are single-stranded non-coding RNAs containing 200 nucleotides. miRNAs consist of 20–24 nucleotides which modulate gene expression via effects of post-transcriptional sort. Over 2000 miRNAs have been spotted in human beings, and they make up one-third of all of the genes found within the genome^(28,44)^. In spite of the aetiological bases of infertility, epigenetic alterations that occur on account of infertility treatment play a role in chronic longer-term diseases, involving differential methylation of genes vital for development and growth with longer-term implications pertaining to health^(28)^. It has not yet been defined if any of the said differentially altered genes are linked to the treatments or to infertility. One study highlighted that antioxidant administration to IVF children enhanced nitric oxide bioavailability and vascular response within systemic as well as pulmonary circulation^(45)^. The results italicise ART-induced vascular dysfunction in young individuals is reversible despite redox regulation. Hence, it is deemed important to be able to come up with a clear perspective pertaining to the factors that come into play in the occurrence of infertility treatment which can improve outcomes^(28)^ (Fig. 1).

### Nutritional models that increase infertility risk

Recently, the key nutrition model pertaining to developed and developing countries has been called the Western diet. The Western diet is portrayed by simple carbohydrates, saturated and trans-fatty acids as well as by a high intake of animal protein alongside essential low unsaturated fatty acids and low dietary fibre^(46)^. The factors affecting the quality of semen have worsened through the spread of this Western diet^(47)^. Processed meats, or as indicated in a number of sources red meat, fatty dairy products, alcohol, sweets and sweet drinks, coffee, potatoes, shortage of whole-grain products, fruits and vegetables, fish and seafood, nuts, poultry and skim dairy products weaken semen parameters and cause low fertility^(48,49)^.

High-fat diet together with obesity caused because of leading an unhealthy impact the nature of spermatozoa, and the development along with the health of the offspring throughout a lifespan. As a matter of fact, dietary patterns of inappropriate sort like inadequate antioxidant intake, high-energy density, also skipping meals have been witnessed in infertile males^(49,50)^. It has been perceived that lower paternal dietary folate can modify the mouse sperm epigenome and to that end is ascribed to adverse outcomes pertaining to pregnancy^(51)^. In the systematic review and meta-analysis, relationships were recognised between the paternal folate status and sperm quality, fertility, congenital malformations placental weight^(52)^. Improper paternal and/or maternal diet may affect the epigenetic marks of the offspring and ultimately lead to infertility^(51–53)^.

#### Phytoestrogens

There is some controversy regarding the effect of phytoestrogens on male reproductive health. Phytoestrogens are plant-derived compounds with estrogen-like activities. The binding affinity of phytoestrogens to estrogen receptors is 100–1000 times lower than estrogen^(54)^. After phytoestrogens bind to the receptors with ligands, they are transported from the cytoplasm to the nucleus, enabling the expression of specific genes. At the same time, since phytoestrogens are in steroid-like structure, they can bind to receptors on the cell surface^(55)^. Due to these properties, phytoestrogens can have an effect on all processes that suppress the synthesis of sex hormone-binding globulin (SHBG) regulated by estrogens and the aromatisation of testosterone^(56)^. Aside from effects of estrogenic sort, it has properties of antimutagenic and antioxidant nature^(57)^. Also, Asian individuals have the capability to convert soy into an estrogen of non-steroidal being by gut bacteria, which is affected by genetic conditioning, diet and the composition of the gut microbiota^(58)^. Existing line of research do not show soybean intake at moderate levels is linked with an elevated risk pertaining to infertility, impaired semen quality alongside decreased levels of blood testosterone. On top of these enhancement in sperm quality components was unveiled in some cases^(59)^. It is known that infertility is seen in sheep consuming high amounts of soy. Although there are studies that associate regular consumption of soy products with decreased sperm count in men and reduced fertility in women, recent studies have reported that consumption of soy products or soy supplement intake will not affect fertility in humans and may even increase the live birth rate with ART^(60–63)^. As a result, the consumption of soy products does not adversely affect fertility and may even reduce the risk of ovulatory infertility in women^(60,62,63)^. However, prospective research investigating the impact pertaining to phytoestrogens is needed to be able to pinpoint the influence of isoflavone consumption in fertility.

#### Antioxidants

In the Cochrane review, in which randomised controlled studies on antioxidant supplementation were published in the treatment of infertility, it was reported that antioxidant supplementation had no benefit in increasing pregnancy or live birth rates^(64)^. Still, selenium deficiency is associated with reproductive system disorders. It is reported that selenium scavenges hydrogen peroxide molecules by decreasing the production of ROS in sperm and increases glutathione peroxidase-1 activity. Daily 400IU vitamin E and 200 μg selenium supplementation for 100 d to infertile men aged 20–45 years resulted in a 52⋅6 % increase in sperm motility and morphology, and 10⋅8 % pregnancy occurred as a result of combined treatment with vitamin E and selenium^(65)^. On the other hand, it is also reported that selenium supplementation alone is not effective in the treatment of infertility. For example, 200–300 mg/d of selenite supplementation, selenium-enriched yeast or high dietary selenium intake did not have a positive effect on sperm characteristics or activity, in spite of increased semen selenium concentrations^(66)^. Another study showed that the combination of 100 μg selenium and 1 mg vitamin A improved sperm motility in subfertile men with low selenium levels. Zinc, another antioxidant trace mineral, is necessary for sperm DNA condensation. The low zinc content of sperm chromatin is associated with male infertility. It is known that selenium and zinc positively affect sperm concentration and sperm motility^(67)^. It has also been suggested that insufficient zinc consumption may damage antioxidant defence and be an important danger factor in oxidant release, and it will be effective in making sperm cells extremely prone to oxidative damage. In men with idiopathic asthenozoospermia or oligospermia, oral zinc supplementation has been found to positively affect sperm motility, morphology and count^(68)^.

#### Vitamins

Vitamin B12 and folate (or folic acid) are under investigation for beneficial effects on fertility. While the effect of defects and folate deficiency in folate as well as homocysteine metabolism on neural tube defects (NTDs) has been determined the evidence for folate's effects on fertility is less clear^(69)^. It is thought that folate may have beneficial effects on fertility in infertility treatment. Folate, which is involved in DNA synthesis, is a very important vitamin for gametogenesis, fertilisation and pregnancy. Thereby, folate (the dietary form) or folic acid (the synthetic form) has a critical role in reproduction^(70)^. Low folate intake is associated with increased anovulation. Increasing the folate stores in the body has been shown to improve oocyte quality during IVF treatment^(54)^. IVF cohort research in Poland discovered females taking folic acid supplements before treatment were found with better quality oocytes as well as with a higher degree of mature oocytes compared to those who did not^(71)^. Another study highlighted that folic acid supplementation in infertile women decreased homocysteine concentrations and increased folate concentrations in follicular fluid, which was associated with greater chances of pregnancy and better embryo quality^(72)^. It is suggested all females of childbearing age take a folic acid supplement of 400 mcg/d and include dark green leafy vegetables which are rich sources of folate, in their diets^(62)^. In a study conducted on subfertile women, it was demonstrated that the probability of getting pregnant was 16 % more in the group taking a multivitamin supplement with 400 mcg/d of folic acid compared to the group taking placebo^(70)^.

Insufficient intake of folate, vitamins B6 and B12, which are influential in the homocysteine pathway in the preconceptional period, is linked to fertility outcomes in partners that are of adverse sort. Insufficient intake of these vitamins can cause an increase in homocysteine levels, generating hyperhomocysteinemia. Evidently, increased homocysteine level in follicular fluid is inversely proportional to oocyte and embryo quality and worsens IVF/ICSI^(54)^.

Vitamins A, C and E (carotenoids, α-tocopherol, ascorbic acid) can provide antioxidant–prooxidant stability and protect the genetic unity of sperm cells by prohibiting oxidative damage in sperm DNA, increase the number of motile sperm as well as sperm count with vitamin C along with B-carotene intake. It has been reported that vitamin C supplement has a positive impact upon stress-induced infertility due to its testosterone-increasing and antioxidant effects. It is also stated that high-dose of vitamin C supplementation increases the testosterone level by affecting the hypothalamus–pituitary–testis axis^(73)^. Antioxidants thought to be associated with infertility are vitamins A, C, E, zinc, selenium, glutathione, coenzyme Q10, carnitine and lycopene. In addition to their positive effects on free radicals, they are also associated with infertility^(74)^. There are vitamin D receptors in the ovaries, uterus, endometrium and placenta. It has been reported that the effect of vitamin D on infertility may also be by increasing the level of interleukin-6 (IL-6) which is one of the pro-inflammatory cytokines. In a study, it was found that women with both low serum vitamin D levels and high IL-6 levels had a 10⋅6 times higher risk of infertility due to tubal factors^(75)^. It has also been pronounced that inflammatory factors such as IL-6 show a significant correlation in individuals with endometriosis complicated with infertility^(76)^.

It has been concluded that there is a direct correlation between semen quality and serum 25(OH) vitamin D levels in men and vitamin D receptors (VDRs) are importantly lower in infertile men compared to other men^(54)^. In adults, low serum vitamin D is also associated with low sperm count, and changes in sperm morphology and sperm motility^(77)^. In another study carried out on infertile women, it was figured out there existed no statistically significant difference between those with and without adequate serum 25(OH) vitamin D levels in terms of follicle-stimulating hormone (FSH) and AMH^(78)^. It is uttered that the beneficial effects of vitamin D on reproductive health occur not only through the VDR in germ cells but also through the ability of other organs in the male reproductive system to express VDR. Vitamin D is also required for transcellular calcium transfer from serum to epididymis, which is required for sperm motility in maturation of spermatoses^(54)^. Antioxidant nutritional supplements such as l-carnitine and CoQ10 can remarkably improve semen parameters. It has been stated that the use of antioxidant nutritional supplements in the treatment of male infertility may be the focus of future reports^(4)^. There are also studies claiming the opposite^(61)^. In another study, it was reported that micronutrient supplementation such as vitamins B6, C as well as D and E, along with folic acid, iron, selenium, and lastly iodine may have a positive effect on infertility treatment^(9)^. Iron, copper and manganese gain a prominent function towards the development of fetus positively impacting female reproductive system. But still, both excess and deficiency of essential trace elements are ascribed to adverse pregnancy outcomes along with infertility in women^(80)^ (Table 1).

**Table 1.** Effects of diet on fertility^(47,62,73,75,78,79)^

Dietary components	Active ingredient	Results
Oily sea fish	PUFA, omega-3 Fat-soluble vitamins A, D, E, K	Fish and seafood are the main sources of DHA and EPA in the diet. So, their inclusion in the diet may be associated with improved semen quality. The negative impact of severe vitamin D deficiency on fertility in women cannot be ignored.
Fruits and vegetables	Minerals, folic acid, antioxidants, fibre	Fruits and vegetables form the basis of healthy eating patterns associated with improving fertility and semen quality. Pesticide residues may alter the favourable effect of vegetable and fruit intake on semen quality. Folic acid affects fertility in women. Doses higher than recommended for the inhibition of neural tube defect and vitamin B1 supplementation might be beneficial. Antioxidant supplements in women do not make a big difference.
Nuts and oilseeds	EFAs, fibre, tocopherols, phytosterols, polyphenols, minerals	It is critical to choose hazelnuts, unroasted and unsalted oil seeds. Consumption of nuts may have a beneficial effect on sperm quality.
Whole-grain products	Fibre, zinc, magnesium	It is recommended to limit the consumption of refined grain products and to prefer whole grain products rich in fibre.
Skimmed milk	Protein, calcium	It is beneficial to choose low-fat dairy products because of their lower saturated fat content.
Olive oil, rapeseed	Polyphenols, vitamin E, alpha-linolenic acid, PUFA	It is recommended to replace saturated fats with vegetable oils containing unsaturated acid residues.

## Conclusions and recommendations

Infertility treatment is time-consuming and costly, as well as an emotionally tiring process. Since there are some non-modifiable factors that determine the success of assisted reproductive technologies, the focus should be on optimising modifiable lifestyle parameters enhancing fertility or the likelihood of the effectiveness of treatment pertaining to infertility. Epigenetic mechanisms can be affected by numerous parameters, namely, aging, environmental influence, dietary energy as well as consumption of trans-fatty and saturated acids, intake of mono and polyunsaturated fatty acids (especially omega 3), animal protein sources, as well as nutritional and non-nutrient compounds, more or less energy intake or methionine interact with D, B12 and B6 vitamins, biotin, choline, iron, selenium, zinc, folic acid, resveratrol and/or quercetin.

Even though the epigenetic mechanisms, genes, nutrition and nutritional supplements discussed in this review have an effect on infertility and the recommended dose has not been determined yet, it has been noted that they can positively affect fertility. However, more comprehensive and longitudinal human studies investigating its relationship with male and female reproductive functions are needed.
